# Motor neuron development in zebrafish is altered by brief (5-hr) exposures to THC (∆^9^-tetrahydrocannabinol) or CBD (cannabidiol) during gastrulation

**DOI:** 10.1038/s41598-018-28689-z

**Published:** 2018-07-12

**Authors:** Kazi T. Ahmed, Md Ruhul Amin, Parv Shah, Declan W. Ali

**Affiliations:** 1 Department of Biological Sciences, CW-405 Biological Sciences Building, University of Alberta, Edmonton, T6G 2E9 Canada; 2 Neuroscience and Mental Health Institute, 4-120 Katz Group Centre, Edmonton, T6G 2E1 Canada

## Abstract

Marijuana is one of the most commonly used illicit recreational drugs and is widely used for medicinal purposes. The psychoactive ingredient in marijuana is ∆^9^-tetrahydrocannabinol (∆^9^-THC), whereas the major non-psychoactive ingredient is cannabidiol (CBD). Here, we exposed zebrafish embryos to ∆^9^-THC or CBD for 5 hours during the critical stage of development known as gastrulation. Embryos were allowed to develop normally and were examined at 2 and 5 days post fertilization. THC and CBD treated embryos exhibited reduced heart rates, axial malformations and shorter trunks. Cannabinoid treatment altered synaptic activity at neuromuscular junctions (NMJs), and fluorescent labelling of primary and secondary motor neurons indicated a change in branching patterns and a reduction in the number of axonal branches in the trunk musculature. Furthermore, there were alterations in the α-bungarotoxin labelling of nicotinic acetylcholine receptors at NMJs. Locomotion studies show that larvae exposed to THC or CBD during gastrulation exhibited drastic reductions in the number of C-start escape responses to sound stimuli, but not to touch stimuli. Together these findings indicate that zebrafish embryos exposed to ∆^9^-THC or CBD during the brief but critical period of gastrulation exhibited alterations in heart rate, motor neuronal morphology, synaptic activity at the NMJ and locomotor responses to sound.

## Introduction

Marijuana is derived from the plant *Cannabis sativa* L. and is commonly used for medicinal purposes^1^. It is a recreational drug^2^ that is often taken along with alcohol^2,3^ and is reported to be the most commonly used illicit drug during pregnancy^4^. Because it is capable of freely crossing the placenta^5,6^, it may pose a significant risk to embryonic development^7,8^. The primary psychoactive ingredient in marijuana is ∆^9^-tetrahydrocannabinol (∆^9^-THC; referred to hereafter as THC), whereas the major non-psychoactive ingredient is cannabidiol (CBD). Links between embryonic exposure to THC and deficits in CNS development have been shown, but significantly less is known about the effects of CBD during development. Unlike THC, CBD lacks psychotropic activity and has been used as an anxiolytic, an appetite stimulant, an analgesic and as a treatment for diseases such as multiple sclerosis and epilepsy^9,10^. Moreover, CBD has been used to reduce bouts of nausea and vomiting during pregnancy^11,12^.

THC binds to and activates 2 distinct classes of G-protein coupled receptors: CB1R and CB2R^13^. CB1Rs are highly localized to the CNS^14–16^ while CB2Rs are primarily associated with the peripheral nervous system and the immune system^17,18^, although recent studies report that CB2Rs are also present in the CNS^19–21^. In chicks and mice, CB1R protein expression first occurs before neuronal development^22^ and increases thereafter in a region-specific manner^23^. In rats, maternal exposure to THC has been linked to altered locomotor and exploratory behavior in their offspring^24^, and in humans, it leads to increased incidences of tremors and startle behaviors^25^. CBD on the other hand has shown limited efficacy at CB1 and CB2Rs and is even thought to behave as an inverse agonist at CB1Rs^26^. It has been suggested to interact with the orphan cannabinoid receptors GPR55 and GPR18, as well as the serotonin 5HT1A receptor and TRP1 channels^27^. How prenatal exposure to THC or CBD affects the development of muscle and motor neurons has not been comprehensively studied.

In this study, we set out to determine if exposure to THC and CBD during zebrafish development, has an effect on cells involved in locomotion. Importantly, we focused our exposure parameters during a period of development known as gastrulation. Gastrulation is a critical stage in embryonic development when the differentiation of cell lines becomes apparent for the first time during embryogenesis. In zebrafish, gastrulation occurs between 5.25 hours post fertilization (hpf) and 10.75 hpf ^28^. Three germ layers- ectoderm, mesoderm and endoderm are formed during this stage, and key neurons including Mauthner cells and primary motor neurons are born. In humans gastrulation occurs in week 3 of embryogenesis^29^ and is early enough that pregnancy may remain undetected.

Zebrafish embryos offer certain distinct advantages over mammalian models for toxicity and exposure studies. Embryos develop outside the mother in a chorion or egg casing, allowing one to accurately control the concentration and the time course of exposure compared with placental animals. Additionally, semi-transparent zebrafish embryos can be used for whole preparation imaging and identifiable neurons can be studied throughout development. Drawbacks include the absence of a maternal-embryo interaction during gestation. But the advantages offered by a zebrafish model for toxicity and teratogenicity are significant and allow for a wide range of studies that may be difficult to perform in other preparations. Here we specifically wanted to determine if a brief pulse of cannabinoids during a key developmental period would alter embryonic development. Our results indicate that heart rate, gross morphology, neuronal branching, synaptic activity and locomotor responses such as the C-start escape response are adversely affected by exposure to THC or CBD.

## Results

### Gross Morphology

**Figure 1:**
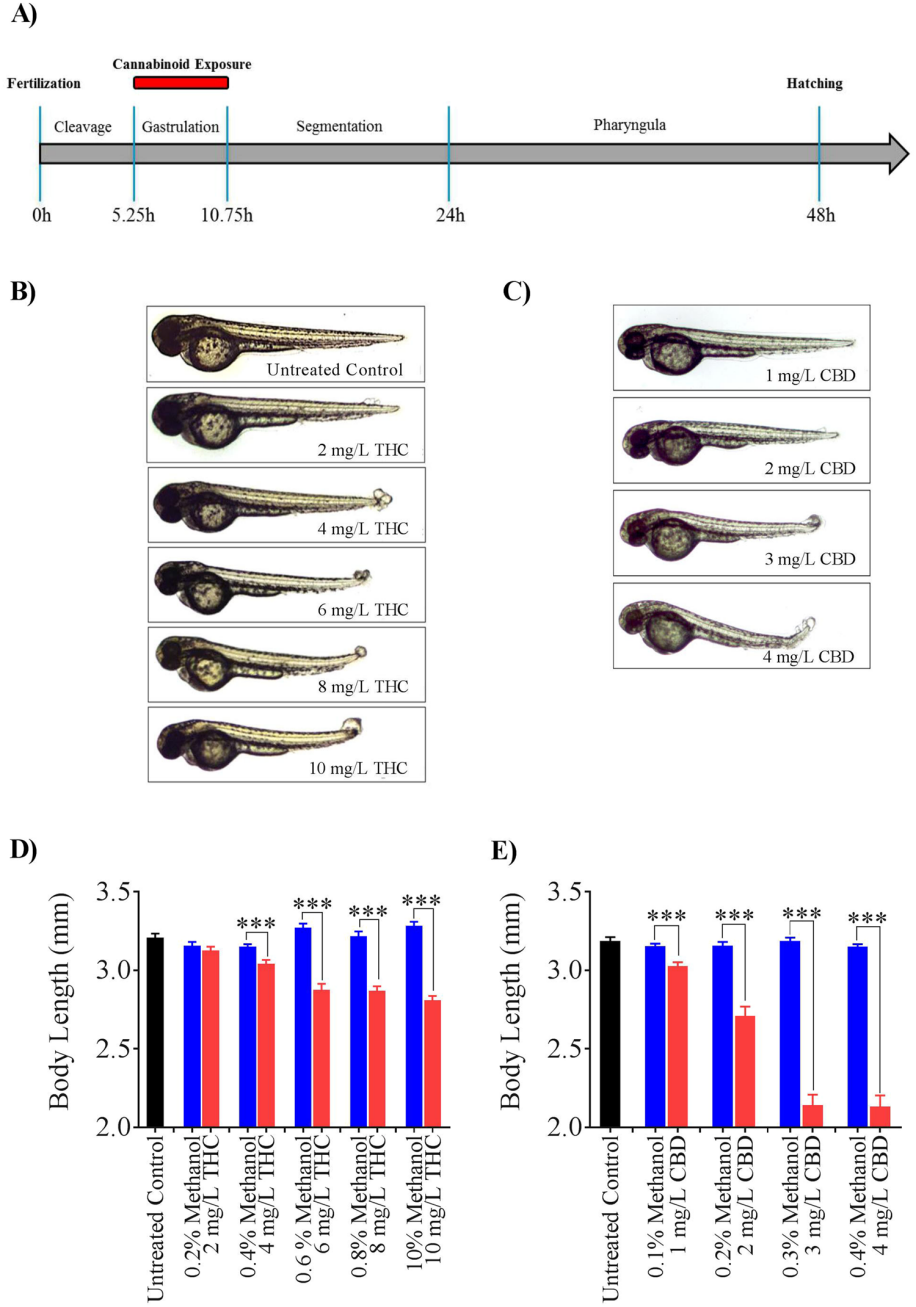
Effect of THC and CBD exposure on zebrafish embryos. (**A**) A schematic of the exposure paradigm of cannabinoids in this study. Red bar shows the duration of the cannabinoid exposure which occurred for 5 hours during gastrulation. (**B**,**C**) Embryos were untreated (control), or exposed to 2 mg/L, 4 mg/L, 6 mg/L, 8 mg/L or 10 mg/L THC or 1 mg/L, 2 mg/L, 3 mg/L or 4 mg/L CBD (from 5.25 hpf to 10.75 hpf and then allowed to develop in normal embryo media. Images were taken at 48–52 hpf. (**D**) Bar graph showing the body lengths of fish in untreated control (black, n = 59), different concentrations of THC (pink, n = 54, 48, 61, 57 and 55 for 2, 4, 6, 8 and 10 mg/L THC-treated fish respectively) or corresponding vehicle control (blue, n = 39, 37, 22, 25 and 20 for 0.2, 0.4, 0.6, 0.8 and 1 percent methanol-treated fish respectively). (**E**) Bar graph showing the body lengths of fish in untreated control (black, n = 51), different concentrations of CBD (pink, n = 52, 52, 25 and 19 for 1, 2, 3 and 4 mg/L CBD-treated fish respectively) or corresponding vehicle control (blue, n = 36, 39, 32 and 37for 0.1, 0.2, 0.3, and 0.4 percent methanol-treated fish respectively). ^***^Significantly different from vehicle control, p < 0.001.

Our goal in this study was to determine if brief exposure to the primary psychoactive and non-psychoactive ingredients in marijuana (THC and CBD) during gastrulation, had adverse effects on embryonic development, specifically focusing on aspects of locomotion. We exposed zebrafish embryos to various concentrations of THC (2, 4, 6, 8 and 10 mg/L), CBD (1, 2, 3 and 4 mg/L) and their vehicle controls (0.1–1% methanol) (Fig. 1A), and examined a range of anatomical features as well as hatching, survival and heart rate. We also examined untreated embryos as additional controls for all treatments (THC, CBD and methanol). The dose-dependent effects on morphology and body length are shown in Fig. 1B,C. Consistent with a previous study, we found that vehicle controls (0.1–1% methanol) had no adverse morphological effects^30^ (Supplemental Figs 1A and 2A). Embryos exposed to increasing concentrations of THC and CBD developed with curved tails, cardiac edema and deformities such as blebbing at the tip of the tail. Additionally, there was a dose-dependent reduction in body length of 2 dpf embryos (Fig. 1D,E). For instance, the mean body length of embryos exposed to 6 mg/L THC was 2.88 ± 0.04 mm (n = 61) compared with vehicle controls of 3.27 ± 0.03 mm (n = 22) (p < 0.001) (Fig. 1D). Similarly, the mean body length of embryos exposed to 3 mg/L CBD was 2.14 ± 0.07 mm (n = 25) compared with 3.19 ± 0.02 mm in vehicle controls (n = 32; p < 0.001) (Fig. 1E).

**Figure 2:**
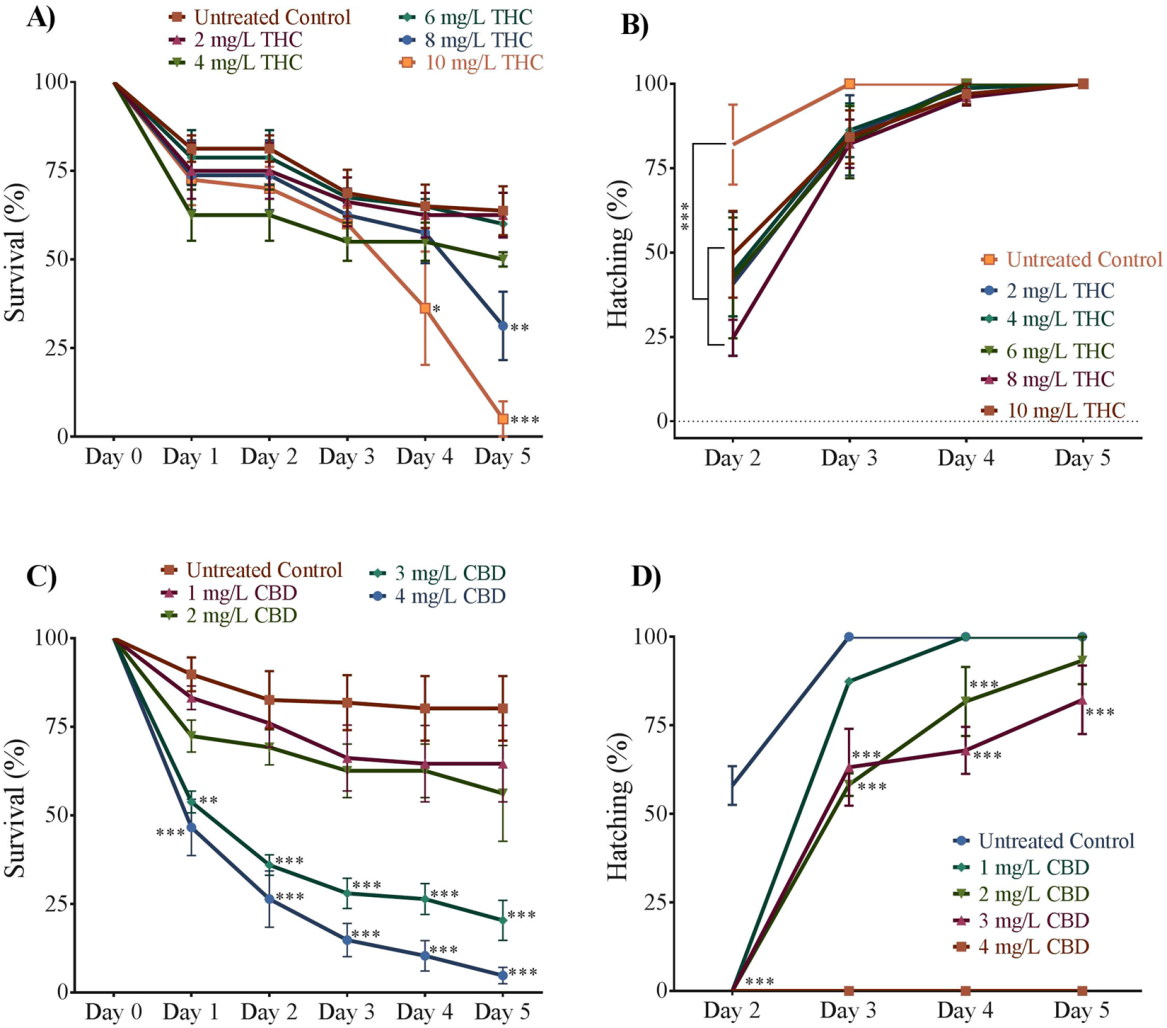
Effect of THC and CBD exposure on survival and hatching. (**A**,**B**) Line graph showing the percentage of embryos that survived and hatched within the first 5 days of development following THC exposure during gastrulation (N = 4 experiments and n = 20 embryos for each treatment). (**C**,**D**) Line graph showing the percentage of embryos that survived (N = 5 experiment and n = 25 embryos for each treatment) and hatched within the first 5 days after egg fertilization following CBD exposure (N = 3 experiment and n = 25 embryos for each treatment). ^**^Significantly different from vehicle control, p < 0.01. ^***^Significantly different from vehicle control, p < 0.001.

To assess survival, we documented mortality rates in the first 5 days of development. Vehicle controls showed no difference in survival from untreated animals (Supplemental Figs 1B and 2B). Embryos exposed to 2–8 mg/L THC experienced similar survival rates for the first 3 days of development (Fig. 2A). By day 5, embryos treated with 8 mg/L THC had a survival rate of only 31 ± 10% (p < 0.05; n = 4 experiments), while embryos treated with 10 mg/L THC only had a 5 ± 5% survival rate (Fig. 2A; p < 0.005; n = 4 experiments). The effects of CBD on survival were more severe. For instance, by day 1 there was only about 47 ± 8% survival in the 4 mg/L treated group (p < 0.01; n = 5 experiments) and 54 ± 3% survival in the 3 mg/L treated group (Fig. 2C; p < 0.01; n = 5 experiments). By 5 days post fertilization, survival rates were 65 ± 11%, 56 ± 14%, 20 ± 6% and 5 ± 2% in the 1–4 mg/L CBD treated groups respectively (Fig. 2C; p < 0.01; n = 5 experiments), compared with ~80% survival in untreated and vehicle controls (Fig. 2C).

Rates of hatching were also negatively impacted by exposure to THC and CBD. In most cases, 100% of untreated animals and vehicle controls hatched by 3 dpf (Fig. 2B,D Supplemental Figs 1C and 2C), whereas only about 75% of THC treated animals (regardless of THC concentration) (Fig. 2B) and 50–90% of CBD-treated animals (87 ± 1% for 1 mg/L, 58 ± 3% for 2 mg/L and 63 ± 11% for 3 mg/L) (Fig. 2D) had hatched. Embryos treated with 4 mg/L CBD did not hatch at any age and died by 5 dpf (Fig. 2D).

**Figure 3:**
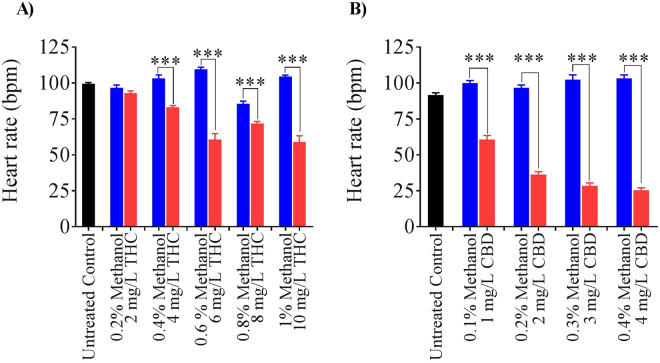
Effect of THC exposure on heart rate. (**A**) Bar graph showing the heart rate of untreated control embryos (n = 29), 2–10 mg/L THC-exposed embryos (n = 26, 22, 22, 21 and 21 for 2, 4, 6, 8, and 10 mg/L THC respectively) and corresponding vehicle controls (methanol-treated embryos; n = 31, 25, 35, 26 and 28 for 0.2, 0.4, 0.6, 0.8, and 1 percent methanol respectively). (**B**) Bar graph showing the heart rate of untreated control embryos (n = 22), 1–4 mg/L CBD-exposed embryos (n = 42, 40, 25 and 20 for 1, 2, 3 and 4 mg/L CBD respectively) and corresponding vehicle treated embryos (n = 25, 31, 24 and 25 for 0.1, 0.2, 0.3 and 0.4 percent methanol respectively). ^***^Significantly different from vehicle control, p < 0.001.

Exposure to THC during gastrulation altered the basal heart rate of 2 dpf embryos compared with vehicle controls (Fig. 3A). Exposure to 2 mg/L THC had no significant effect on heart rate, but embryos treated with 4 mg/L THC exhibited heart rates that were lower than controls (Fig. 3A), while exposure to 6, 8 and 10 mg/L THC significantly reduced heart rates by up to 50% (Fig. 3A; p < 0.001; n = 21–22). The heart rate of untreated embryos was 99 ± 1 (n = 26) beats per minute. Embryos treated with 2 mg/L THC exhibited a heart rate of 93 ± 2 (n = 26) beats per minute, while embryos treated with 4, 6, 8 and 10 mg/L THC had heart rates of 83 ± 1 (n = 22), 61 ± 4 (n = 22), 72 ± 1 (n = 21) and 59 ± 4 (n = 21) respectively (Fig. 3A). Embryos treated with CBD also exhibited a dose-dependent decrease in heart rate. For instance, embryos treated with 1, 2, 3 and 4 mg/L CBD had heart rates of 61 ± 3 (n = 42), 36 ± 2 (n = 40), 29 ± 2 (n = 25) and 25 ± 2 (n = 20) beats per minute respectively, compared with controls that ranged from 97 ± 11 to 103 ± 12 beats per minute (Fig. 3B; p < 0.001). These data indicate that a 5-hour exposure to THC and CBD during gastrulation significantly lowers the heart rate of newly hatched zebrafish embryos. For the remainder of the study we treated embryos with single concentrations of THC and CBD (6 mg/L THC and 3 mg/L CBD) because these concentrations were in the 30–80% range for hatching and survival of 2 dpf embryos.

### Electrophysiology

**Figure 4:**
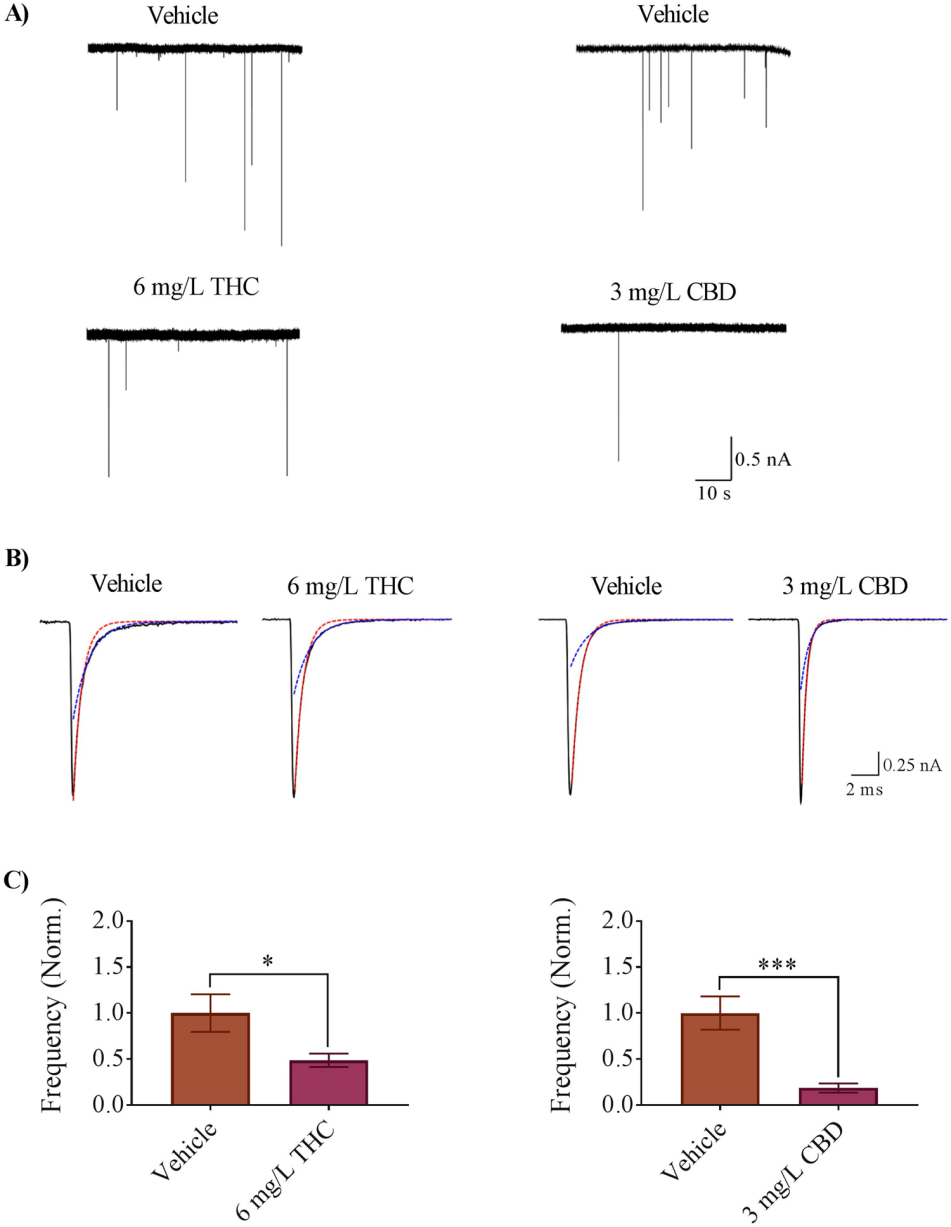
Miniature endplate currents (mEPCs) recorded from zebrafish white muscle fibers of vehicle control and 6 mg/L THC-treated embryos (left column), and vehicle control and 3 mg/L CBD-treated embryos (right column). (**A**) Raw traces obtained from 2 dpf vehicle control (0.6% Methanol, 0.3% Methanol), 6 mg/L THC-treated embryos and 3 mg/L CBD treated embryos. (**B**) Averaged mEPCs obtained from white muscle (black line) fit with a single exponential decay over the fast component (τ_fast_, red dashed line) or slow component (τ_slow_, blue dashed line). Averaged mEPCs acquired from vehicle control (0.6% Methanol 6 events; 0.3% Methanol 36 events), 6 mg/L THC-treated embryos (11 events) and 3 mg/L CBD (6 events) (**C**) Bar graph of the mean mEPC frequency of vehicle and THC-treated embryos (left), and vehicle and CBD-treated embryos (right). ^*^Significantly different from vehicle control, p < 0.05. ^***^Significantly different from vehicle control, p < 0.001.

One of our goals was to determine if exposure to THC and CBD during gastrulation altered the activity of locomotor systems, specifically focusing on the NMJ. Therefore, we asked whether synaptic activity at the NMJ was affected by exposure to cannabinoids. To investigate this, we recorded miniature endplate currents (mEPCs) from the white fibers associated with trunk musculature. Embryonic zebrafish have two types of muscle fibers, tonic red fibers and twitch white fibers that are easily identifiable under the microscope. We particularly focused on white fibers as mammalian skeletal musculature is mostly comprised of twitch fiber types. In zebrafish, white fibers make up the bulk of the trunk musculature and are innervated by both primary and secondary motor neurons^31^. We only analyzed the fast rise time mEPCs in our recordings since these events occurred on the cells we were recording from rather than from neighboring, electrically-coupled cells. The frequency of the fast rise time mEPCs recorded from vehicle controls was 0.1 ± 0.01 Hz (n = 9), whereas in the THC (6 mg/L) treated embryos the mEPC frequency was reduced by almost 50% to a value of 0.04 ± 0.006 Hz (n = 8) (p < 0.01; Fig. 4A,C), and in the CBD treated animals, it was 0.02 ± 0.01 Hz (n = 9) compared with vehicle controls (0.13 ± 0.02, n = 10) (p < 0.001). In fact, in some preparations we recorded only 2–4 mEPCs over a 4-minute time period. There was no change in mEPC amplitude compared with vehicle controls (p < 0.05; data not shown).

We had previously found that the decay time course of mEPCs recorded from white fibers of 2 dpf embryos was bi-exponential in nature due to the presence of multiple classes of nAChRs^32,33^. Because changes in the kinetics often signify a change in the subunit composition of synaptic receptors, we examined the exponential decay of mEPCs but found that there was no significant difference amongst any of the treatments (Fig. 4B). These data imply that the nAChR subtypes that are normally expressed at zebrafish NMJs are not altered by exposure to THC or CBD.

### Motor neuron immunolabelling

**Figure 5:**
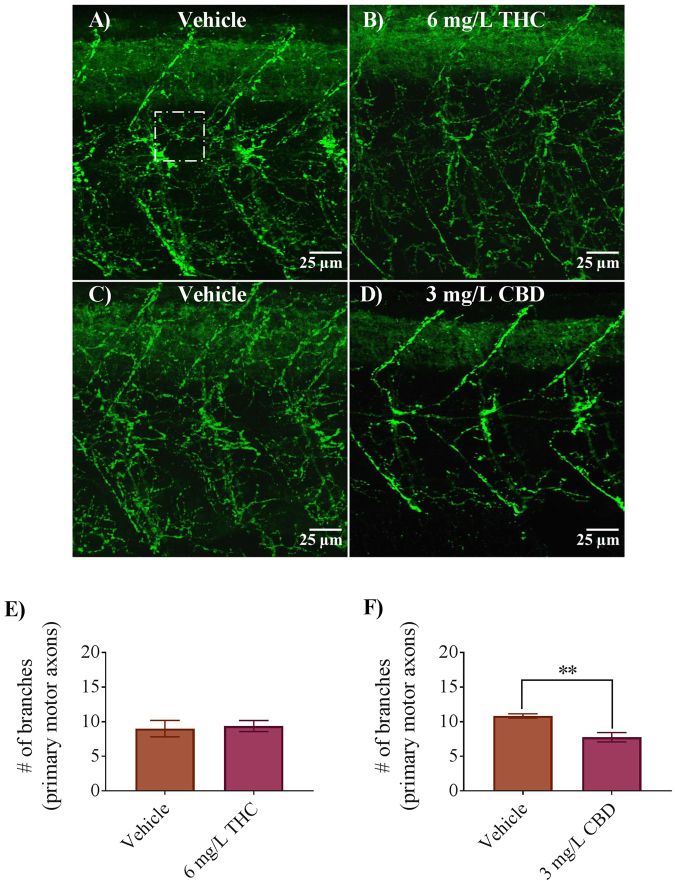
Antibody labelling (anti-znp1) of axonal branches of primary motor neurons in 2 dpf embryos in vehicle controls, 6 mg/L THC-treated embryos and 3 mg/L CBD treated embryos. (**A**–**D**) Branching patterns and labelling of axons appear to be similar between controls and THC-treated embryos but reduced in CBD treated embryos. (**E**) Bar graph showing the number of branches emanating from primary motor axons in vehicle control (n = 7) and 6 mg/L THC treated embryos (n = 8), counted from 9 different square areas (each 1500 μm^2^ area). (**F**) Bar graph showing the number of branches emanating from primary motor axons in vehicle control (n = 6) and 3 mg/L CBD treated embryos (n = 8), counted from 9 different square areas (each about 1500 μm^2^ area). ^*^Significantly different from vehicle control, p < 0.01.

Our electrophysiological data suggested that activity at zebrafish NMJs was affected by exposure to THC and CBD. A reduction in mEPC frequency usually indicates a change in presynaptic properties, therefore we asked whether the morphology of primary and secondary motor neurons was altered by THC or CBD treatment. To determine motor neuron innervation patterns, we immunolabelled primary and secondary motor neurons with anti-znp1 and anti-zn8 respectively. The anti-znp1 antibody recognizes a form of synaptotagmin 2 that is present in zebrafish primary motor neurons^34,35^, while the anti-zn8 antibody recognizes DM-GRASP protein that is highly localized to the cell membranes of secondary motor neurons^35,36^. First, we examined the axons of primary motor neurons labelled with anti-znp1 and found that exposure to 6 mg/L THC had no quantifiable effect on the number of branches emanating from the main, primary axon (Fig. 5A,B), whereas exposure to 3 mg/L CBD resulted in a significant reduction in the number of axonal branches (Fig. 5C,D). Specifically, we found that the number of branches (per 1500 µm^2^ square area; Fig. 5A) was reduced from a mean value of 11 ± 1 (n = 6 embryos) in vehicle controls to 8 ± 1 (n = 8 embryos) in CBD-treated animals (Fig. 5F, p < 0.01).

**Figure 6:**
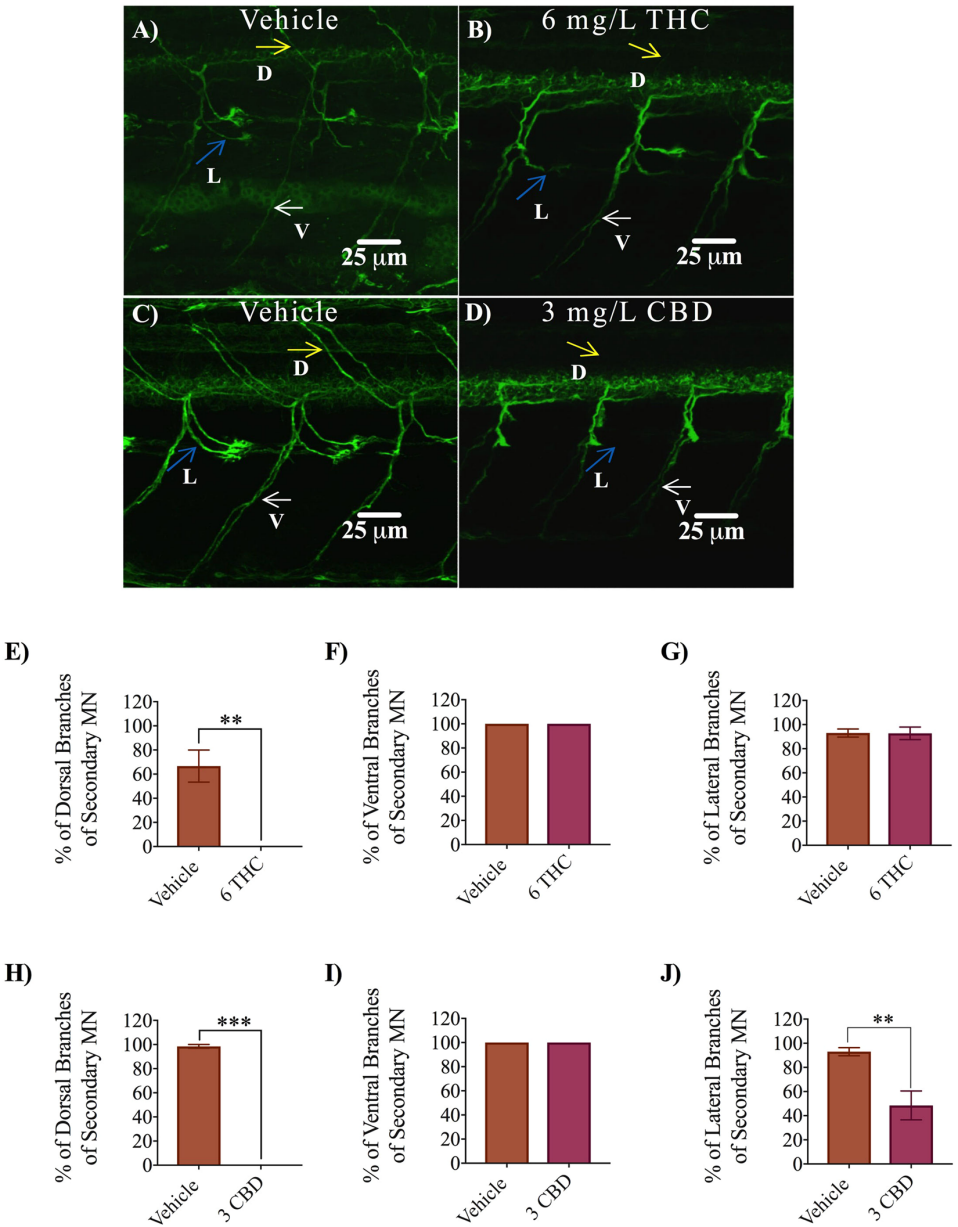
Antibody labelling (anti-zn8) of axonal branches of secondary motor neurons in 2 dpf embryos in vehicle control, 6 mg/L THC-treated embryos and 3 mg/L CBD treated embryos. (**A**–**D**) Dorsal, ventral and lateral branches emanating from secondary motor neurons are indicated by yellow, white and blue arrows. Dorsal branches were absent in THC and CBD treated embryos (**B**,**D**). Fewer lateral branches are visible in CBD treated embryos. (**E**–**G**) Bar graph comparing percentage of dorsal branches (**E**), ventral branches (**F**) and lateral branches (**G**) emanating from secondary motor neurons in vehicle control (n = 11) and 6 mg/L THC treated embryos (n = 11). (**H**–**J**) Bar graph comparing percentage of dorsal branches (**H**), ventral branches (**I**) and lateral branches (**J**) emanating from secondary motor neurons in vehicle control (n = 11) and 3 mg/L CBD treated embryos (n = 9). ^**^Significantly different from vehicle control, p < 0.01. ^***^Significantly different from vehicle control, p < 0.001.

Immunolabelling of secondary motor neurons with anti-zn8 showed a more severe effect of THC and CBD exposure, compared with primary motor neurons. The nature of the fluorescent labelling allowed us to specifically quantify dorsal, ventral and lateral branching patterns, as shown in Fig. 6 (labelled D, V and L). We were unable to see dorsal branches of secondary motor neurons in embryos treated with either 6 mg/L THC or 3 mg/L CBD (Fig. 6B,D,E,H; p < 0.01), whereas ventral branches were always present but often looked thinner than controls (Fig. 6B,D, F and I). Lateral branches were usually present in THC treated animals but were largely absent following CBD treatment (Fig. 6B,D,G and J; p < 0.01). Taken together, these data suggest that the normal development and innervation patterns of primary and secondary motor neurons were affected by exposure to THC and CBD during gastrulation.

### nAChR labeling

**Figure 7:**
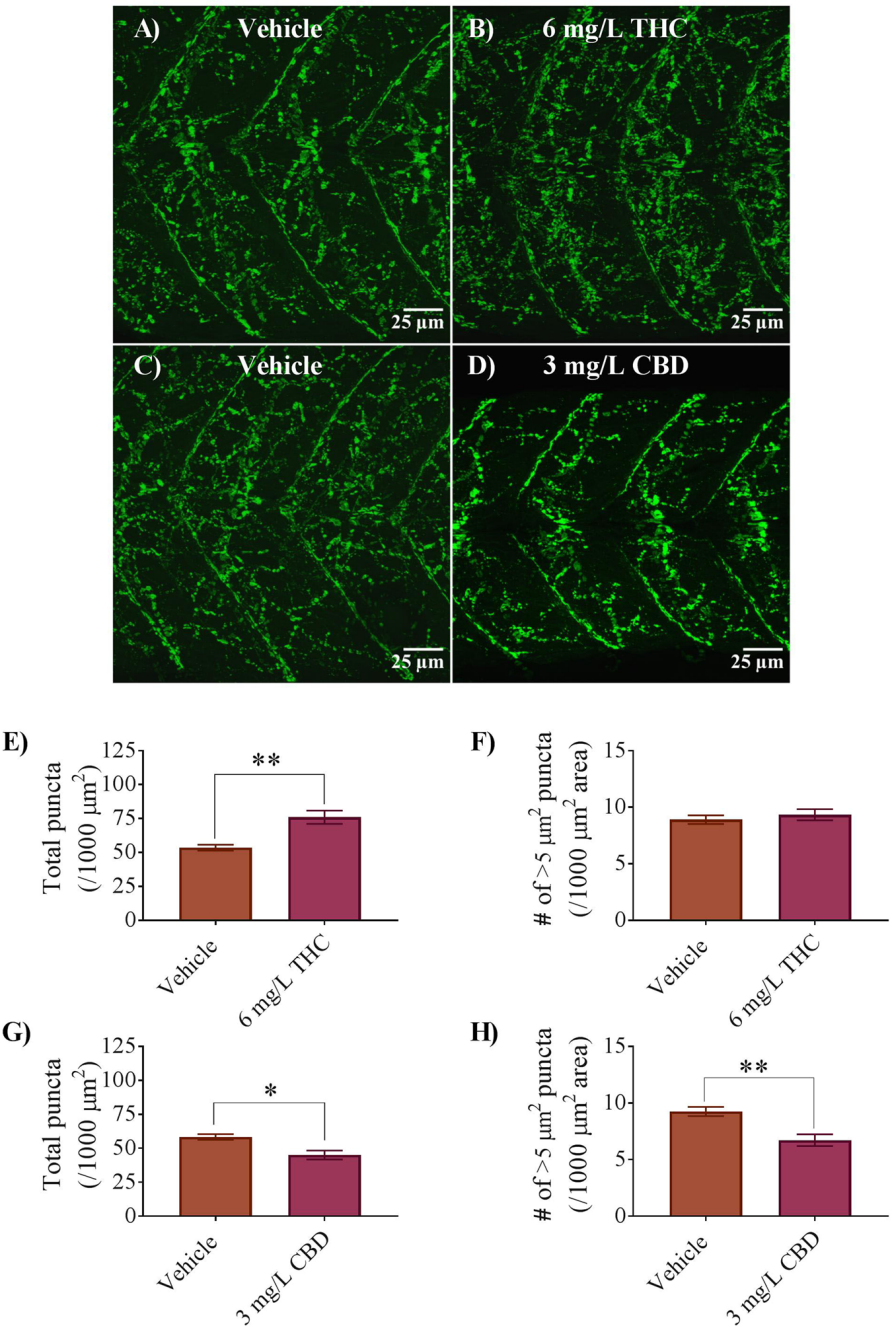
Expression of nicotinic acetylcholine receptors (nAChRs) in 2 dpf embryos of controls, 6 mg/L THC and 3 mg/L CBD-treated fish. (**A**–**D**) α-bungarotoxin labelling of postsynaptic membranes at the NMJ in zebrafish trunk musculature. (**E**) Bar graph representing the total number of α-bungarotoxin puncta counted over per 1000 μm^2^ area and compared between vehicle control (n = 10) and 6 mg/L THC-treated embryos (n = 6). (**F**) Bar graph representing the number of α-bungarotoxin puncta with a minimum area of ~5 μm^2^, compared between vehicle control (n = 10) and 6 mg/L THC-treated embryos (n = 6). (**G**) Bar graph representing the total number of α-bungarotoxin puncta counted over a 1000 μm^2^ area and compared between vehicle control (n = 8) and 3 mg/L CBD-treated embryos (n = 7). (**H**) Bar graph representing the number of α-bungarotoxin puncta with a minimum area of ~5 μm^2^, compared between vehicle control (n = 8) and 3 mg/L CBD-treated embryos (n = 7). * Significantly different from vehicle controls, p < 0.05. **Significantly different from vehicle controls, p < 0.01.

To determine if the expression of nicotinic acetylcholine receptors (nAChRs) at NMJs was altered by THC and CBD exposure, we used fluorescently tagged α-bungarotoxin to label postsynaptic membranes of neuromuscular junction NMJs (Fig. 7). The fluorescent labelling of NMJs consistently appeared brighter in the THC treated embryos compared with controls. The total number of (nAChR) puncta was greater in THC treated embryos compared with vehicles by approximately 22% (p < 0.05; Fig. 7A,B and E). In contrast, there was a 13% reduction in the total number of nAChR puncta in CBD treated embryos (Fig. 7C,D and G). The size of the puncta ranged from a minimum of less than 1 μm^2^ to a maximum of 300 μm^2^. The smallest puncta appeared to be discrete entities and may represent extrasynaptic nAChRs or developing endplates, whereas large puncta (>10 μm^2^) likely represent mature NMJ clusters. Further analysis revealed that there were fewer puncta with a minimum size of ~5 μm^2^ in CBD treated animals compared with controls (Fig. 7D,H). These findings suggest that synaptic development at the NMJ was altered following cannabinoid treatment during gastrulation.

### Locomotion

**Figure 8:**
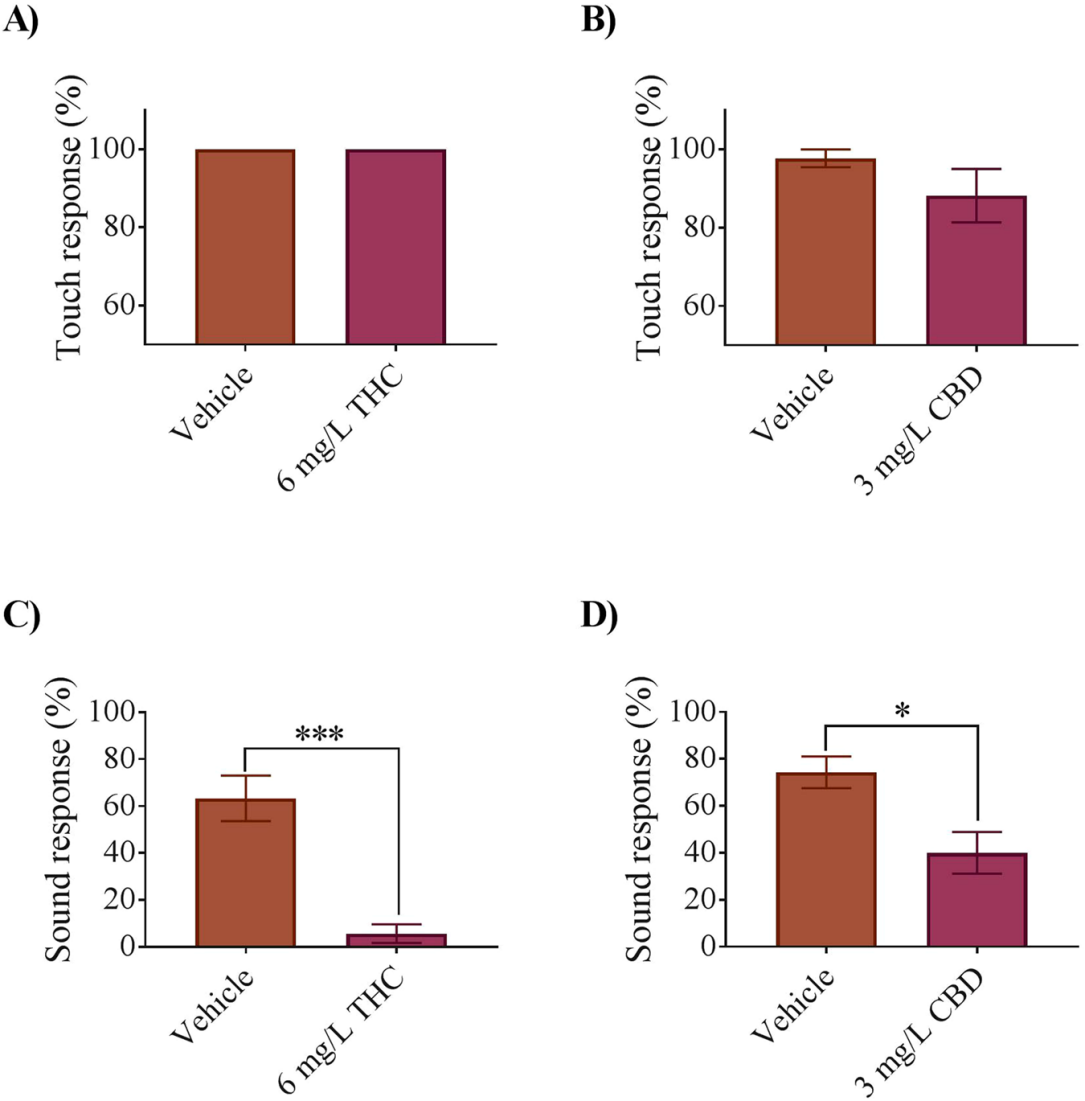
Quantification of the response rate of 5-dpf zebrafish larvae to touch and sound stimuli. (**A**) Bar graph comparing the ratio of larvae responding to a touch stimulus in vehicle control (n = 39 embryos in 4 experiments) and 6 mg/L THC (n = 51 embryos in 4 experiments). (**B**) Bar graph comparing the ratio of larvae responding to a touch stimulus in vehicle control (n = 42 embryos in 4 experiments) and 3 mg/L CBD (n = 25 embryos in 3 experiments) (**C**) Bar graph comparing the ratio of larvae responding to a sound stimulus in vehicle control (n = 30 embryos in 5 experiments) and 6 mg/L THC (n = 30 embryos in 5 experiments). (**D**) Bar graph comparing the ratio of larvae responding to a sound stimulus in vehicle control (n = 24 embryos in 4 experiments) and 3 mg/L CBD (n = 30 embryos in 5 experiments). ^*^Significantly different from controls p < 0.05. ^***^Significantly different from controls p < 0.001.

Cannabinoid exposed embryos were able to swim (data not shown). To determine if exposure to cannabinoids altered their ability to respond to stimuli such as touch or an acousto-vestibular input, we stimulated free-swimming 5 dpf larvae with a mechanical or sound stimulus since the AV input onto Mauthner cells develops at 4 dpf. Vehicle control larvae responded to touch about 100% of the time (Fig. 8A,B). Embryos treated with THC or CBD also showed strong touch response rates of 100% (n = 51 fish in 4 experiments) and 88% respectively (Fig. 8A,B) (n = 25 fish in 3 experiments). However, the response to sound was very different. Vehicle control larvae responded approximately 68% of the time and following THC treatment exhibited a drastic reduction in response where only 6% responded to the sound pulse (n = 30 fish in 5 experiments) (Fig. 8C). CBD treated embryos only responded at a rate of 40% (n = 30 fish in 5 experiments) when given a sound stimulus. These findings show that motor systems are still functional following cannabinoid treatment, but there appears to be a selective effect on different sensory modalities.

Taken together these results suggest that cannabinoid treatment during the 5-hour time period of gastrulation altered a number of characteristics in developing zebrafish embryos including morphology, heart rate, activity at the neuromuscular junctions, MN branching and ability to respond to sound stimuli.

## Discussion

Here we show that zebrafish embryos exposed to THC and CBD for 5 hours during gastrulation exhibited physical abnormalities by the time of hatching, alterations in motor neuron branching and reduced C-start escape responses when compared with untreated and vehicle-treated embryos. Our most significant findings can be summarized as follows: embryos treated with THC and/or CBD exhibited 1) shorter body lengths and mild deformities, 2) reduced survival, 3) reduced heart rates (up to 50% reduction), 4) decreased frequency of mEPC activity at the NMJ, 5) alterations in branching patterns of secondary MNs, 6) changes in the expression of postsynaptic nAChRs associated with skeletal musculature and 7) reduced response rates to sound stimuli. Thus, our results suggest that exposure to THC and CBD very early in life may alter embryonic development.

THC is the main psychoactive ingredient in the plant, *Cannabis sativa*. Cannabis has been characterized as the most commonly used illicit drug in pregnant women^37^ with about 5% of pregnant women reporting drug use on a monthly basis. Moreover, in North America, there has been an increase in the marijuana use in women of reproductive age^38^, and an increase in the potency and content of THC in marijuana in the last 25 years^39^. Cannabis may be used by pregnant women to reduce morning sickness, while CBD has been used to treat patients with nausea and loss of appetite during cancer therapies^11,12^. The recent legalization of Cannabis in various parts of world including several jurisdictions in Canada and the United States, has highlighted the paucity of information on the effects of cannabinoids during very early development. Thus, it is important to understand the effects of cannabis exposure on developing embryos. We chose to study cannabinoid exposure at a key time point in development, gastrulation, which occurs very early in embryogenesis. An important aspect of our study is that we focused on a brief period of exposure, for only ~5 hours, rather than chronic exposure over long term. Under these conditions, we find that exposure to THC and CBD alters embryonic development. For some of our experiments we chose to use concentrations of THC and CBD (6 mg/L and 3 mg/L respectively) that we believe to be roughly within the physiological range of cannabis use in humans. Blood plasma concentrations of THC can peak as high as 0.25 mg/L during the smoking of a single cigarette^40^. In our study, we exposed embryos to a concentration of 2–10 mg/L while the newly fertilized eggs were still in the chorion, or egg casing. Under these conditions, approximately 0.1–10% of toxicants typically cross the chorion^41,42^, suggesting that approximately 0.006–0.6 mg/L was directly exposed to the embryo. Moreover, the THC content of marijuana has increased several-fold over the last 10–15 years. The CBD content in marijuana also varies tremendously, but when used for medicinal purposes is often used in concentrations that range from 5 mg/kg to as high of 100 mg/kg^43^, administered intra-peritoneally, with a daily maximum dose of ~1500 mg/day^44^.

We found a significant reduction in heart rate following exposure to THC and CBD at almost all concentrations used in our dose response studies. The acute effects of cannabinoids on the resting heart rate of adult organisms are known to be inconsistent and may lead to no change, an increase or a decrease in heart rate^45–47^. In our study, we exposed embryos during gastrulation and then examined the developmental effects at later stages. Zebrafish cardiac progenitor cells are present as early as the 512-cell stage in the early blastula^48^, and during gastrulation these precardiac cells involute, turn towards the animal pole and reach the embryonic axis around the 8-somite stage, where they combine to form two myocardial tubes^48^. Therefore, at the time of exposure, cardiac progenitor cells are present and may be impacted by THC and CBD treatment.

We examined activity at the NMJ by recording mEPCs from fast twitch skeletal muscle of 2-day old embryos. We specifically analyzed the fast rise time events in our recordings since these events are due to synapses located on the cells we were recording from rather than from neighboring, electrically-coupled muscles^49^. The primary effect of THC and CBD was a reduction in mEPC frequency which typically signifies the presence of fewer synaptic sites, or a change in the release characteristics from motor neurons. Because the mEPC amplitude and decay properties were not affected by THC or CBD treatment, we focused our attention on presynaptic effects such as the morphology and branching patterns of motor neurons. Immunolabelling showed that exposure to CBD significantly altered the branching of spinal motor neurons, whereas the effect of THC was less severe at the concentrations we tested. Zebrafish white skeletal muscle fibers are innervated by a single primary motor neuron and multiple secondary motor neurons. Primary motor neurons are born around 9–11 hpf and their axons pioneer a path out to skeletal muscle^31^. In each hemi-segment there are 3 (sometimes 4) primary motor neurons and up to 20–24 secondary motor neurons. The primary motor neurons have undergone their final round of DNA synthesis starting at 9 hpf, while the secondary motor neurons first appear about 5–6 hours later^50^. Therefore, primary motor neuron cell bodies are present at the time of exposure and may be directly impacted by cannabinoids. Secondary motor neurons are not born until at least 5–6 hours following exposure and yet they are still significantly affected by THC and CBD. Previous studies on zebrafish have shown that downregulation of the endocannabinoid 2-AG induces weaker swimming performances^51^ while a 96-hour exposure to THC and CBD induces hypo-locomotor activity^52^. Cannabinoids are highly lipophilic substances and may actually remain associated with cell membranes long after the exposure time frame has elapsed. If so, then the effects of cannabinoids may continue long after direct exposure has ended.

What is the mechanism of action of THC and CBD in gastrulating zebrafish? THC and CBD may act on a number of different receptors including, but not limited to the CB1Rs and CB2Rs, GPR55 and GPR18, as well as serotonin receptors 5HT1Rs and vanilloid TRPV1 receptors^13,26,27^. CBD may even act as an inverse agonist at CB1 and CB2Rs. The typical cannabinoid receptors (CB1R and CB2R) are present from the earliest stages of neuronal life and in the developing chick the CB1Rs first appear in the CNS as early as the birth of the first neurons^53^. In embryonic organisms CB1 agonists and antagonists are capable of altering axonal growth^54^, and signaling through the endocannabinoid system has been shown to play chemo-attractive and chemo-repulsive roles in developing cortex^55,56^. Several reports provide strong evidence for an interaction between the endocannabinoid system and growth factors during early development. For instance, in cerebellar neurons CB1R activation linked to FGF receptor activity influences neurite outgrowth, while CB1R interaction with TrKB receptors in cortical interneurons is required for interneuron migration and specification^55^. Thus, the endocannabinoid system has the ability to control neuronal migration and differentiation by regulating growth factor activity. The endocannabinoid system has also been shown to modulate the expression of neurotransmitters in the basal ganglia that are involved in movement such as GABA and glutamate^19^. Thus, our results of altered neuronal branching and changes in locomotion are consistent with previous findings.

Despite small changes to the development of motor neuron innervation patterns and the control of trunk muscle fibers, zebrafish embryos and larvae were still capable of responding to mechanical stimuli with fairly robust C-starts. However, they were largely incapable of responding to sound. Moreover, the effects were more pronounced following THC treatment compared with CBD treatment. The fact that a response to sound was vastly diminished may implicate an impairment of hair cell function in cannabinoid treated animals. Inner hair cells in mammals express CB2 receptors, while outer hair cells show significantly reduced expression^57,58^. Thus, precocious activation of CB2Rs on precursor hair cells may impact or even delay normal development, leading to impaired sound detection. More work needs to be done to understand these effects.

One of the most interesting aspects of our study is the time course of the actions of the cannabinoids which were applied for only 5 hours during gastrulation. The effects that we have noted occurred well beyond the exposure time period and suggest that brief exposure may have far-reaching consequences. While our results should be interpreted with care, more research clearly needs to be done to fully understand the impact of cannabinoid exposure on developing organisms. Our findings suggest that even brief exposure may have an impact on embryonic health and development.

## Methods

### Animal care and exposure to THC

The fish used in this study were wild type zebrafish (*Danio rerio*) embryos of the Tubingen Longfin (TL) strain that were maintained at the University of Alberta Aquatic Facility. All animal housing and experimental procedures in this study were approved by the Animal Care and Use Committee at the University of Alberta (AUP #00000816) and adhered to the Canadian Council on Animal Care guidelines for humane animal use. For breeding, 3 to 5 adults, usually consisting of 3 females and 2 males, were placed in breeding tanks the evening before eggs were required. The following morning, fertilized eggs were collected from the breeding tanks, usually within 30 mins of fertilization. Embryos and larvae were housed in incubators on a 12 h light/dark cycle, and set at 28.5 °C. Embryos were exposed to egg water (EW; 60 mg/ml Instant Ocean) containing either THC (2, 4, 6, 8 and 10 mg/L diluted from a stock solution obtained from Sigma; ∆^9^-Tetrahydrocannabinol solution 1.0 mg/ml in methanol) or CBD (1, 2, 3 and 4 mg/L diluted from a stock solution obtained from Sigma; CBD solution 1.0 mg/ml in methanol), or equivalent amounts of methanol during the period of gastrulation, which occurs between 5.25 hpf to 10.75 hpf. The exposure medium was then replaced at 10.75 hpf with 25 mL of fresh EW. Embryos were washed several times in EW and then incubated in fresh EW until further experiments at 48 hpf. For immunohistochemical studies, pigment formation was blocked by adding 0.003% phenylthiourea (PTU) dissolved in egg water at 24 hpf. All protocols were carried out in compliance with guidelines described by the Canadian Council for Animal Care (CCAC) and the University of Alberta.

### Embryo imaging and morphological observations

Embryos were imaged at 2 dpf using a Lumenera Infinity2-1R color microscope camera mounted on a dissecting microscope. Embryos were placed in a 16-well plate with one embryo per well and were anesthetized in 0.02% MS222. Morphological observations were performed using a dissecting microscope. Measurements of embryo length were done using a microscope eyepiece equipped with a micrometer.

### Immunohistochemistry

Embryos (2 dpf) were fixed in 2% paraformaldehyde for 1–2 h and washed with 0.1 M phosphate buffered saline (PBS) every 15 minutes for 2 hours. The preparations were then permeabilized for 30 min in 4% Triton-X 100 containing 2% BSA and 10% goat serum. Tissues were incubated for 48 hours at 4 °C in either mouse monoclonal anti-znp-1 which targets an isoform of synaptotagmin 2 that is highly localized in zebrafish primary motor axons^34,35^, or mouse monoclonal anti-zn-8^35^ (DSHB), which targets the DM-GRASP protein on the surface of secondary motor axons^36,59^. All primary antibodies were diluted at 1:250 in PBS. Tissues were washed in PBS twice every 15 minutes for 2–3 hours and then incubated for 4 hours at room temperature in the secondary antibody, Alexa Fluor® 488 goat anti-mouse IgG, (Molecular Probes, Life Technologies), at a dilution of 1:1000. The embryos were then washed for 7 h with PBS and mounted in MOWIOL mounting media. For labelling of nAChRs, embryos at 2 dpf were permeabilized as previously stated and incubated with 100 nM Alexa-488 conjugated α-bungarotoxin (Molecular Probes, Invitrogen) for 4 hours at room temperature. Embryos were then washed for 7 h with PBS and mounted in MOWIOL mounting media. All embryos were imaged on a Zeiss LSM confocal microscope and photographed under a 40 × objective. Images were compiled using Zeiss LSM Image Browser software and are shown as maximum intensity z-stack compilations. For primary motor axon branches, 9 square boxes (Fig. 5A; each about 1500 μm^2^ area) were evenly placed over the trunk (3 in dorsal, 3 in middle and 3 in lateral regions) and the number of branches per square box were counted and averaged. Image J was used to quantify the axonal branching, and number and size of α-bungarotoxin puncta.

### Electrophysiology

Whole-cell patch clamp recordings were taken from muscle cells of embryos at 2 dpf. Patch-clamp electrodes were pulled from borosilicate glass (GC150T; World Precision Instruments, Sarasota, FL, USA) on a P-97 pipette puller (Sutter Instrument Co., Novato, CA, USA) and fire-polished (Micro-Forge MF-830; Narishige, Japan); once filled with intracellular solution, these tips had series resistances of 2–4 MΩ. The intracellular solution (ICS) consisted of (mM): 130 CsCl, 8 NaCl, 10 Hepes, 10 EGTA, 2 CaCl•2H_2_O, 4 Mg-ATP, 0.4 Li-GTP; the pH was adjusted to 7.4 and osmolarity was adjusted to 290 ± 2 mOsmol l^-1^. An extracellular solution (ECS), which consisted of (mM): 134 NaCl, 2.9 KCl, 1.2 MgCl, 10 Hepes and 10 glucose, with an osmolarity of 280 ± 2 mOsmol L^-1^, adjusted to pH 7.8, was bubbled with air and continuously washed over the preparation, starting ≥ 5 minutes prior to recording. The ECS contained the voltage-gated Na^+^ channel blocker tetrodotoxin (TTX; Tocris, UK) at a concentration of 1 μM in order to block action potentials during mEPC recordings. White muscle fibers were easily and accurately identified based on their orientation within each segment using Nomarski Differential Interference Contrast (DIC) optics, and whole cell voltage-clamp recordings were taken over periods of 1 minute. Whole cell currents were recorded at a holding potential of -60 mV using an Axopatch 200B amplifier (Axon Instruments, Sunnyvale, CA, USA), low-pass filtered at 5 kHz and digitized at 50 kHz. Once in the whole cell recording mode, the fibers had series resistances from 3–6 MΩ. Synaptic currents were recorded in 1-minute epochs. After each 1-minute recording the series resistance was checked and if it had changed by more than 20%, the recording was aborted. Recordings were maintained as long as the membrane resistance remained greater than 10 × the series resistance. Series resistances were compensated by 70% using the amplifier’s compensation circuitry.

### Analysis of mEPCs

Miniature endplate currents (mEPCs) were monitored using a Macintosh iMac computer running AxoGraph X v1.1.1 software (Axon Instruments). Recordings were examined by the software, and synaptic events were detected using a template function. Overlapping or misshapen events were removed and the remaining events were averaged and the properties (amplitudes, decay time constants, frequencies) of the averaged trace were recorded. Events with slow rise times and low amplitudes originate from neighbouring, coupled cells and were excluded from the analysis, therefore, only fast rise time events were included in our analysis since these events originated from the cells we were patch clamping rather than from nearby, electrically-coupled muscles^49^. Single decay time constants were fit over the initial (fast) decay portion and over the distal (slow) portion of the decay. For each *n*, currents were recorded from a single red or white muscle fiber from a single embryo.

### Locomotor response

To image the zebrafish escape response, we used a high speed AOS video camera (AOS S-PRI 1995; 1250 FPS; shutter speed: 800 μs) mounted on a dissecting microscope^60^. For the sound stimulus six larvae, aged 5 dpf, were placed in 35 mm × 10 mm petri dishes with embryo media and were allowed to acclimate to their environment for 30 minutes prior to sound stimulus application. The sound stimulus was a sawtooth waveform (500 Hz, 95–100 dB), created using audacity software (version 2.2.1). A computer speaker was positioned next to the petri dishes to deliver auditory/ vibrational (AV) stimulation to embryos. Escape responses were recorded immediately prior to delivering the stimulus and then for about 1000 ms following the stimulus. This period of time was long enough to film the escape response and periods of swimming following the C-bend. The locomotion to an auditory pulse was scored as an escape response when the animal began the characteristic C-bend after the stimulus.

For the touch response larvae 10–12 were placed in a 35 mm × 10 mm petri dish and were allowed to acclimate to their environment prior to application of the mechanical stimulus. The mechanical stimulus consisted of a light touch to the head with a pair of forceps. C-starts were captured with the AOS high speed video camera.

### Statistics

All values are reported as means ± SEM (standard error of the mean). Significance was determined using a non-parametric t-test between vehicle and treated group followed by Mann-Whitney analysis where appropriate (p < 0.05). Comparisons between multiple groups were done by one-way ANOVA followed by a Tukey post-hoc multiple comparisons test. Statistical analysis was done using the statistical software built in to GraphPad prism.

## Electronic supplementary material

Supplementary Information
