# Supplementary material for: Motor neuron development in zebrafish is altered by brief (5-hr) exposures to THC (∆9-tetrahydrocannabinol) or CBD (cannabidiol) during gastrulation
Source: Sci Rep. 2018 Jul 12;8:10518. doi: 10.1038/s41598-018-28689-z (PMC6043604; doi:10.1038/s41598-018-28689-z)
Supplement: Supplementary file 1 — Supplementary Information [file 41598_2018_28689_MOESM1_ESM.docx]

**Supplementary Information**

**Motor neuron development in zebrafish is altered by brief (5-hr) exposures to THC (Δ^9^-tetrahydrocannabinol) or CBD (cannabidiol) during gastrulation**

Kazi T. Ahmed^1*^, Md Ruhul Amin^1*^, Parv Shah

and Declan W. Ali^1,2^

**Institutional Affiliation:** ^1^Department of Biological Sciences and

Physiology, and the ^2^Neuroscience and Mental Health Institute

CW-405 Biological Sciences Building

University of Alberta

Edmonton, Alberta, Canada

T6G 2E9

*Contributed equally to the study

**Running title:** neurodevelopment in zebrafish is altered by THC and CBD

**Corresponding Author:** Declan W. Ali

Department of Biological Sciences

CW-405, Biological Sciences Building

University of Alberta

Edmonton, Alberta, Canada

T6G 2E9

Phone: 1.780.492.6094

Fax: 1.780.492.9234

Email: declan.ali@ualberta.ca

**Key Words:** cannabinoids, motor neurons, muscle, NMJ, CNS

**
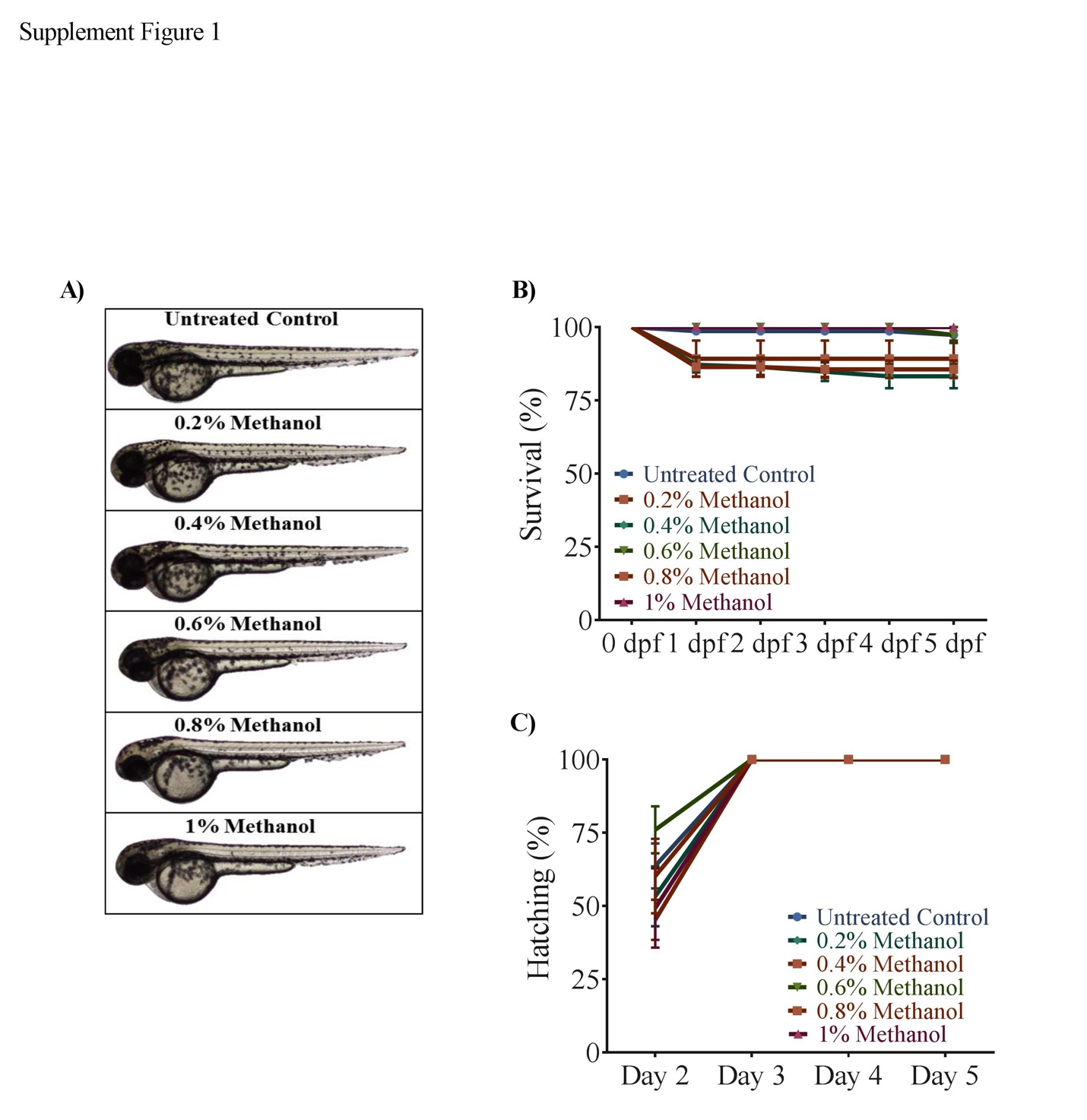
**

**
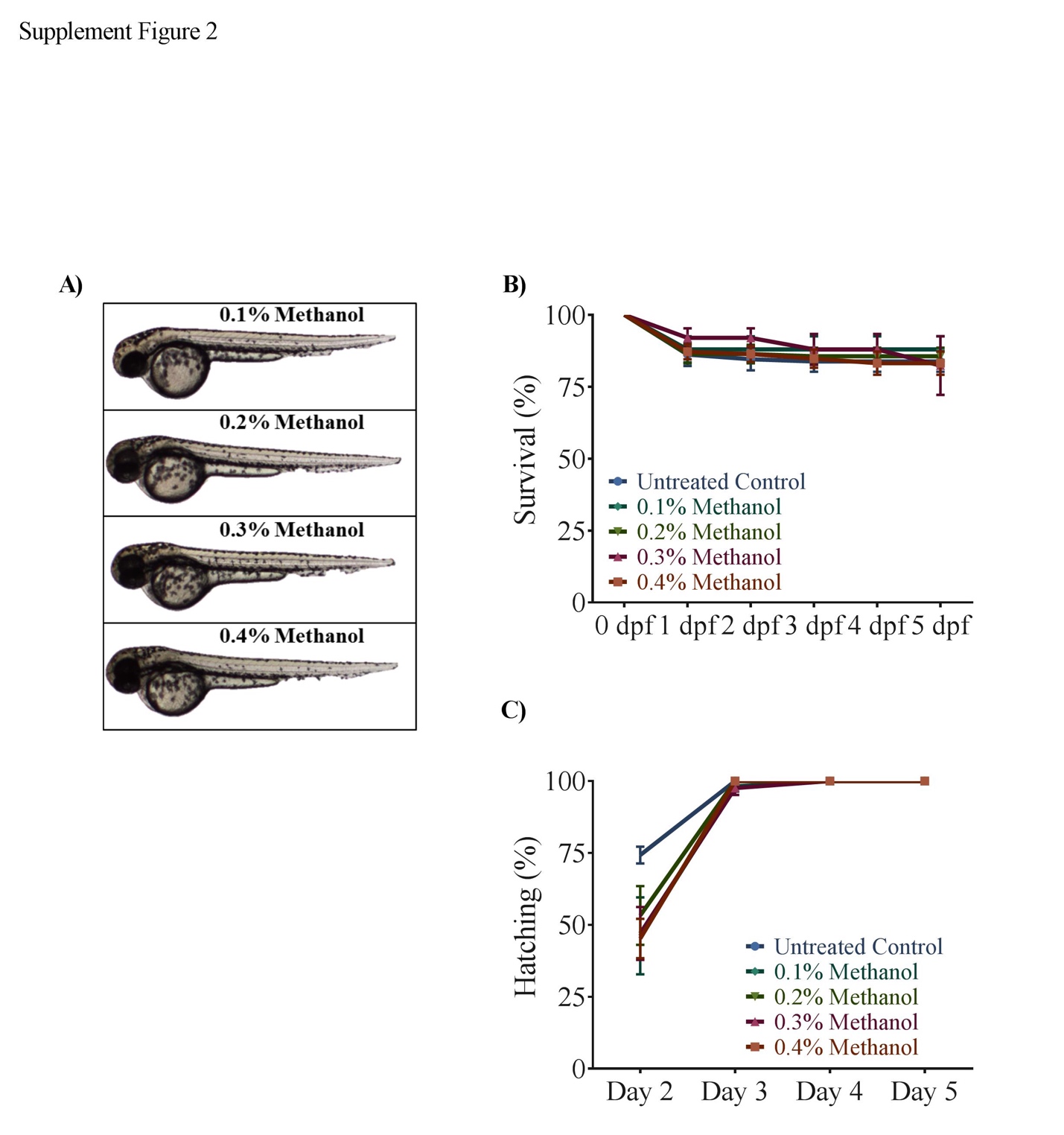
**

**Supplement Figure 1.** Effect of vehicle control of THC (methanol) on morphology, survival and hatching of zebrafish embryos. (A) Embryos were untreated (control), or exposed to 0.2%, 0.4%, 0.6%, 0.8% or 1% methanol (from 5.25 hpf to 10.75 hpf) and then allowed to develop in normal embryo media. Images were taken at 48-52 hpf. (B) Line graph showing the percentage of embryos that survived within the first 5 days of development following methanol exposure during gastrulation (N=5 experiments and n=125 embryos for each treatment). (C) Line graph showing the percentage of embryos that hatched within the first 5 days after egg fertilization following methanol exposure (N=3-5 experiment and n=75-125 embryos for each treatment).

**Supplement Figure 2.** Effect of vehicle control of CBD (methanol) on morphology, survival and hatching of zebrafish embryos. (A) Embryos were exposed to 0.1%, 0.2%, 0.3% or 0.4% methanol (from 5.25 hpf to 10.75 hpf) and then allowed to develop in normal embryo media. Images were taken at 48-52 hpf. (B) Line graph showing the percentage of embryos that survived within the first 5 days of development following methanol exposure during gastrulation (N=5 experiments and n=125 for each treatment). (C) Line graph showing the percentage of embryos that hatched within the first 5 days after egg fertilization following methanol exposure (N=5 experiments and n=125 embryos for each treatment).
